# Structural Design and Analysis of Hybrid Drive Multi-Degree-of-Freedom Motor

**DOI:** 10.3390/mi13060955

**Published:** 2022-06-16

**Authors:** Zheng Li, Hui Zhao, Xuetong Chen, Shenhui Du, Xiaoqiang Guo, Hexu Sun

**Affiliations:** 1 School of Electrical Engineering, Hebei University of Science and Technology, Shijiazhuang 050018, China; zhaohuihbkd@163.com (H.Z.); chenxuetong2020@163.com (X.C.); dushenhui@hebust.edu.cn (S.D.); 2 School of Electrical Engineering, Yanshan University, Qinhuangdao 066004, China; gxq@ysu.edu.cn

**Keywords:** hybrid drive, piezoelectricity, electromagnetism, torque

## Abstract

Piezoelectric-driven multi-degree-of-freedom motors can turn off self-lock, withstand high and low temperatures, are small in size and compact in structure, and can easily achieve miniaturization. However, they have a short life cycle and limited applications. In addition, high-intensity operation will result in a decrease in their stability. Electromagnetic-driven multi-degree-of-freedom motors, on the other hand, are simple and highly integrated, but they are large in volume and lack positioning accuracy. Therefore, combining the two drive modes can achieve complementary advantages, such as improving the motor’s torque, accuracy, and output performance. Firstly, the structure of the hybrid drive motor is introduced and its working principle is analyzed. The motor can achieve single and hybrid drive control, which is beneficial to improving the performance of the motor. Secondly, the influence of magnetization mode, permanent magnet thickness, slot torque, and stator mode on the motor is analyzed. Thirdly, the structure of the motor is determined to be 6 poles and 15 slots, the thickness of the permanent magnet is 12 mm, and the radial magnetization mode is used. Finally, the mixed torque and speed of the motor in the multi-degree-of-freedom direction are tested by experiments, which indirectly verifies the rationality of the structure design.

## 1. Introduction

It has been decades since the piezoelectric motor was originally proposed by Soviet scholars, but its true transition from theory to application has caused an upsurge in research by many scholars, which has developed rapidly in a matter of nearly 20 years. It uses the characteristics of piezoelectric materials to transform energy and output mechanical energy. It is widely used in aerospace, precision processing, and other fields because of its low speed, large torque, small size, convenient control, power-off self-locking, low-noise operation, etc. Electromagnetic-driven motors have been around for a long time. They have mature control principles, wide output range, and high integration, and are widely used in many situations. Piezoelectric and electromagnetic drive methods also have corresponding drawbacks. Piezoelectric-driven motors generally have a shorter service life and are not suitable for continuous operation, and they require a higher driving voltage. Electromagnetic-driven motors have a complex structure, low control accuracy, and are not ideal on precision occasions. Therefore, considering the combination of the two drive modes, a hybrid drive motor is designed, which can make full use of the advantages of each drive mode and avoid the drawbacks of both. This motor has high speed and high thrust under electromagnetic drive, long service life, high dynamic response under piezoelectric drive, high control precision, and passive self-locking. In recent years, many new structural motors with multiple degrees of freedom have been proposed by domestic and foreign scholars, which have had good output performance.

J. Wang et al. introduced the development of a new spherical motor that provides multi-coordinate motion in a driving module. It is based on a spherical rotator and guides in a sphere, just like a ball. Therefore, three-dimensional motion can be performed. The permanent magnet on the rotor and the electromagnetic coil on the stator are controlled by a single power converter, respectively, to achieve high torques at all locations [1]. Li Xuerong et al. proposed a design scheme of a spherical motion generator based on a spherical parallel robot. The new motion generator integrates the electromagnetic actuator with a coaxial 3-RRR spherical parallel robot, which makes the structure more compact and lighter, and has the advantages of having no gaps, high stiffness, and low inertia [2].

Zhang Xiaofeng and others designed and manufactured a new robot finger joint based on a hybrid multi-degree-of-freedom piezoelectric ultrasonic motor. The motor stator is composed of a multi-layer piezoelectric longitudinal vibrator and a sandwiched bending vibrator. Bending vibration can be excited in two orthogonal directions under phase voltage. The combination of longitudinal vibration and bending vibration enables the spherical rotor to rotate in three directions [3]. Based on the principle of inertial drive, Huang Kuanyu et al. developed a motor with multi-axis rotation and precise angle position control. The actuator is composed of three biaxial shear driving units made of D15 piezoelectric shear plates, which can realize multi-degree-of-freedom rotation by using friction to drive the spherical rotor. A magnet is used to generate magnetic attraction preload to make the stator and rotor contact stably [4]. 

Lin Hongyun et al. designed a piezoelectric–electromagnetic hybrid vibration generator with high output power, which can be used in wireless sensor nodes and self-powered microelectronics [5]. R Okeya et al. combined an electromagnetic motor with an ultrasonic motor in order to design a force feedback device that exhibits elasticity, hardness, and roughness [6].

In this paper, a new type of hybrid drive multi-degree-of-freedom motor is proposed, which combines the piezoelectric and electromagnetic driving modes. Through theoretical analysis and simulation software, the performance of the motor is tested through experiments, and the rationality of the motor structure design is verified. The output performance of this motor is better than the motor [7] that relies on two different piezoelectric modes that need to be excited at the same time. Compared with [8], this motor leveraged both electromagnetic and piezoelectric advantages.

## 2. Structure and Working Principle

### 2.1. Hybrid Drive Motor Structure

**Figure 1:**
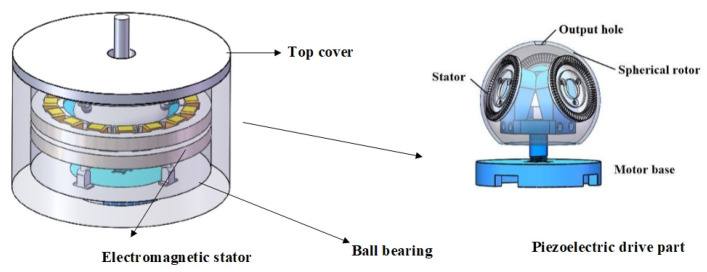
Hybrid drive structure.

As shown in Figure 1, the electro-piezoelectric hybrid drive motor is composed of a built-in piezoelectric drive structure and an external electromagnetic drive structure. 

The piezoelectric drive structure consists of a piezoelectric stator, a spherical rotator, a pre-pressure device, an output shaft, and a motor base, which can be used as a single ultrasonic motor. The electromagnetic drive structure is composed of two layers of electromagnetic stator, a top cover, a ball bearing, and a permanent magnet. The motor can be driven by a single piezoelectric or electromagnetic drive to make the motor move with multiple degrees of freedom, and it can also be controlled by a hybrid drive to improve the performance of the motor. The electromagnetic part of the stator is a fractional-slot winding and the slot type is a semi-closed flat-bottom slot. The pole logarithm of the motor (2*p* = 6) is large so that the thickness of the stator yoke can be reduced appropriately. In doing so, the motor volume can be reduced, the pole distance can be shortened, the end of the winding can be saved, and the copper consumption of the winding can be reduced. The ball bearings are designed to support the spherical rotors and reduce their weight. The permanent magnets are spherical and have six poles, and are uniformly attached to the spherical rotors. The main structure parameters of the motor are listed in Table 1.

### 2.2. Working Principle

Ultrasonic motors utilize the reverse piezoelectric effect of piezoelectric ceramics so that they operate in d31 mode; any two pieces of ceramic in close proximity with different polarization directions will cause them to develop two phases with opposite deformation in tension and contraction, respectively. The elastomer of the stator does not allow it to produce this kind of stretching distortion, but, instead, produces a transverse bending distortion. This distortion is stimulated by mutual alternation, which results in a bending vibration of the stator base. This vibration will exist as a standing wave when only one voltage is applied to it.

The knotless circular mode is used to make the rotator rotate uniformly along the tangential direction of the circle driven by the particle on the stator surface, that is, B_0n_. The motor is designed in B_09_ mode, with nine waves on each traveling wave-type stator. Given the mode function of spatially different π/2 between phases *A* and *B*, the standing wave equation of phase *A* piezoelectric ceramics is:

(1) $$W_{A}\left(r,\theta ,t\right)=\xi_{A}\sin n\theta \cos\omega_{n}t$$

The B-phase standing wave equation is:

(2) $$W_{B}\left(r,\theta ,t\right)=\xi_{B}\cos n\theta \cos\left(\omega_{n}t+\alpha \right)$$

where $\xi_{A}$ and $\xi_{B}$ are the amplitudes of A and B phase stators, respectively, and α is the spatial phase difference. When two standing waves overlap and α=π/2, $\xi_{A}=\xi_{B}=\xi_{0}$, the following positive traveling wave equation is obtained:

(3) $$W\left(r,\theta ,t\right)=\xi_{0}\sin\left(n\theta -\omega_{n}t\right)$$

**Figure 2:**
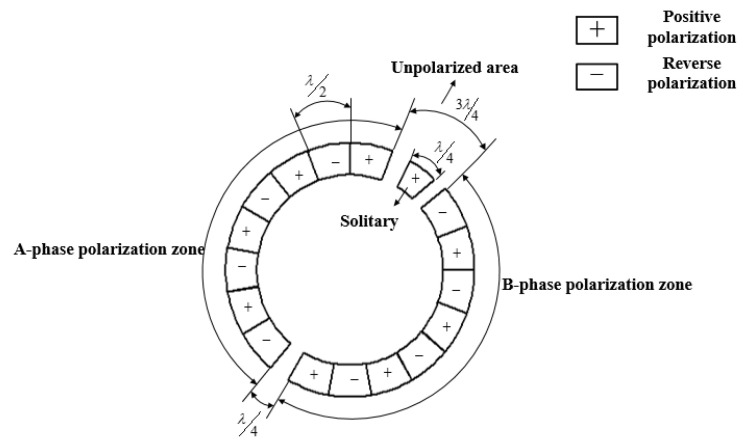
Schematic diagram of piezoelectric ceramic sheet.

The piezoelectric ceramics are divided into two polarized regions as shown in Figure 2, phase A and phase B. The length of the single chip is λ/2, and the radians are π/9, 3λ/4, and λ/4, which are separated as unpolarized regions. λ/4, as a solitary region, has no excitation effect on the stator, and there is no voltage excitation in this region, but it is positively polarized. It utilizes the positive piezoelectric effect of the piezoelectric ceramics. When the traveling wave is generated, the solitary region will also produce a vibration, which serves as the feedback pole for the frequency tracking signal. Under the action of the positive piezoelectric effect, traveling waves are stimulated to produce vibration, which can be used as feedback poles to track frequency signals. To further control the precise deflection of the rotor, a closed-loop design can be added here [9].

**Figure 3:**
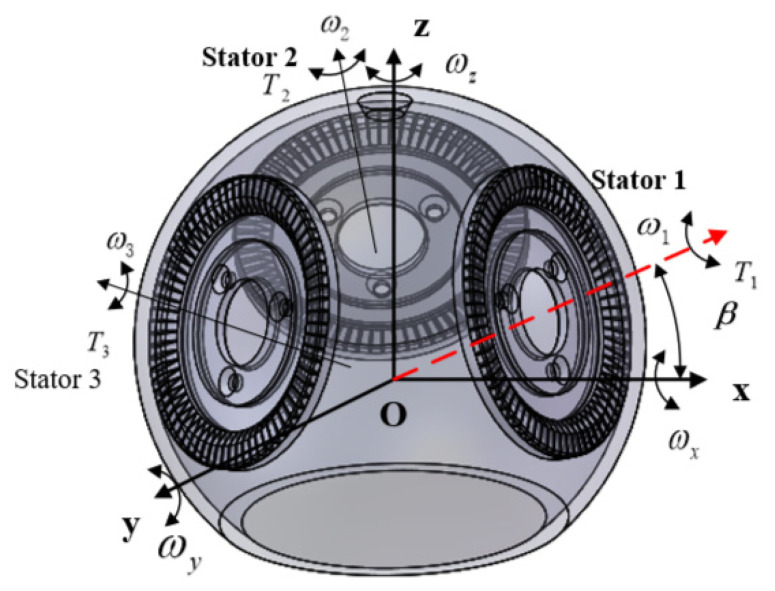
Torque distribution diagram of piezoelectric internal structure.

According to Figure 3, assuming that the central axes $\omega_{x}$, $\omega_{y}$, and $\omega_{z}$ of three piezoelectric stators rotate around the *x*, *y*, and *z* axes, respectively, the angular velocity is:

(4) $$\omega_{s}=\left[\begin{array}{l} \omega_{x}\\ \omega_{y}\\ \omega_{z} \end{array}\right]=\left[\begin{array}{l} \frac{\sqrt{3}}{2}\omega_{1}\cos\alpha_{1}-\frac{\sqrt{3}}{2}\omega_{2}\cos\alpha_{1}\\ -\frac{1}{2}\omega_{1}\cos\alpha_{1}+\omega_{2}\cos\alpha_{1}-\frac{1}{2}\omega_{3}\cos\alpha_{1}\\ \omega_{1}\sin\alpha_{1}+\omega_{2}\sin\alpha_{1}+\omega_{3}\sin\alpha_{1} \end{array}\right]$$

The output torque of the superimposed ultrasonic motor is:

(5) $$T=\left[\begin{array}{l} T_{1}\cos\alpha_{1}-\frac{1}{2}\left(T_{2}+T_{3}\right)\cos\alpha_{1}\\ \frac{\sqrt{3}}{2}\left(T_{2}-T_{3}\right)\cos\alpha_{1}\\ \left(T_{1}+T_{2}+T_{3}\right)\sin\alpha_{1} \end{array}\right]$$

**Figure 4:**
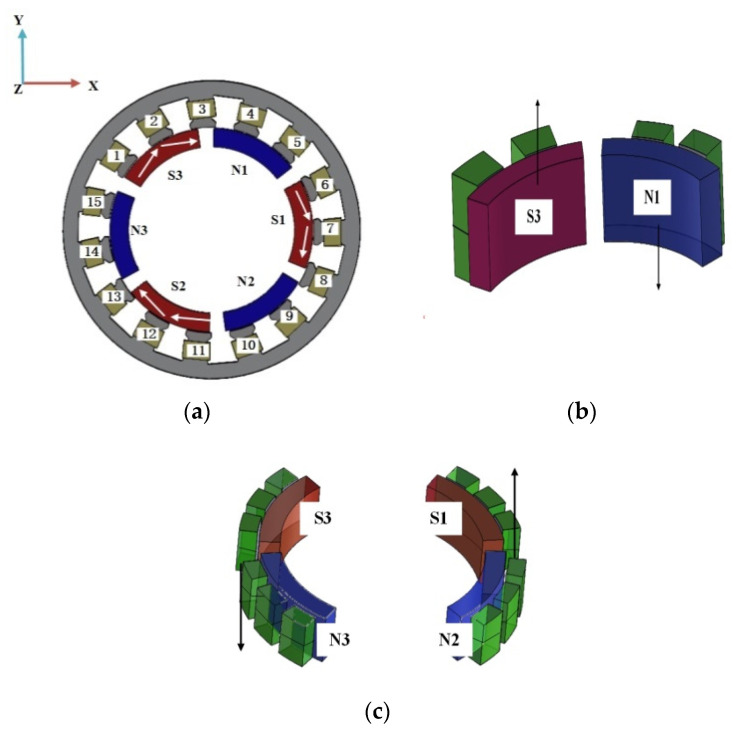
Electromagnetic section working principle: (**a**) *z*-axis positive turn; (**b**) *x*-axis deflection; and (**c**) *y*-axis deflection.

As shown in Figure 4, the coils are marked clockwise and the coils of the two layers of stators are arranged in the same order. The three pairs of permanent magnets are numbered according to N1, S1; N2, S2; and N3, S3, respectively.

According to Ampere’s rule and the principle that magnets are equal to each other and opposite to each other, a current with the same size and direction as coils 1 and 1′ is applied to generate the S-pole magnetic field, which has an all-directional repulsion force on S3. A current with the same size and direction as 2, 2′, 3, and 3′ is applied to excite the N-pole magnetic field and generate tangential attraction to S3. The S-pole magnetic field is generated for 6′, 6′, 7′, 8′, 8′, and 8′, which also makes S1 subject to tangential repulsion and attraction. Similarly, the S-pole magnetic field is generated for coils 11 and 11′, and the N-pole magnetic field is generated for coils 12, 12′, and 13′. Together with these attractions and repulsions, the motor can rotate around the *z*-axis to achieve self-rotation.

When 4′, 5′, 1, and 2 are powered on, the S-pole magnetic field is generated. When 4, 5, 1′, and 2′ are powered on, the N-pole magnetic field is generated; that is, the attraction and repulsion force will force N1 and S3 down. When 9, 10, 11′, and 12′ are powered on, the S-pole magnetic field is generated. When 9′, 10′, 11, and 12 are powered on, the N-pole magnetic field is generated. When the attraction and repulsion force produced by the powered coil come into effect, N2 and S2 move the motor up. This synthesizes electromagnetic torque due to the interaction of clockwise tangential forces on the *x*-axis, which enables the motor to deflect steadily on the *x*-axis.

Similarly, the N-pole magnetic field is generated when 6, 7, 8, 1′, 2′, 3′, 9′, 10′, 14, and 15 are powered on, and the S-pole magnetic field is generated when 6′, 7′, 8′, 1, 2, 3, 9, 10, 14′, and 15′ are powered on. This makes S1 and N2 drive the motor upward under the action of force, while S3 and N3 drive the motor downward, so that the motor can operate stably around the *y*-axis at an angle.

## 3. Analysis of Air Gap Magnetic Density

### 3.1. Magnetization Method

**Figure 5:**
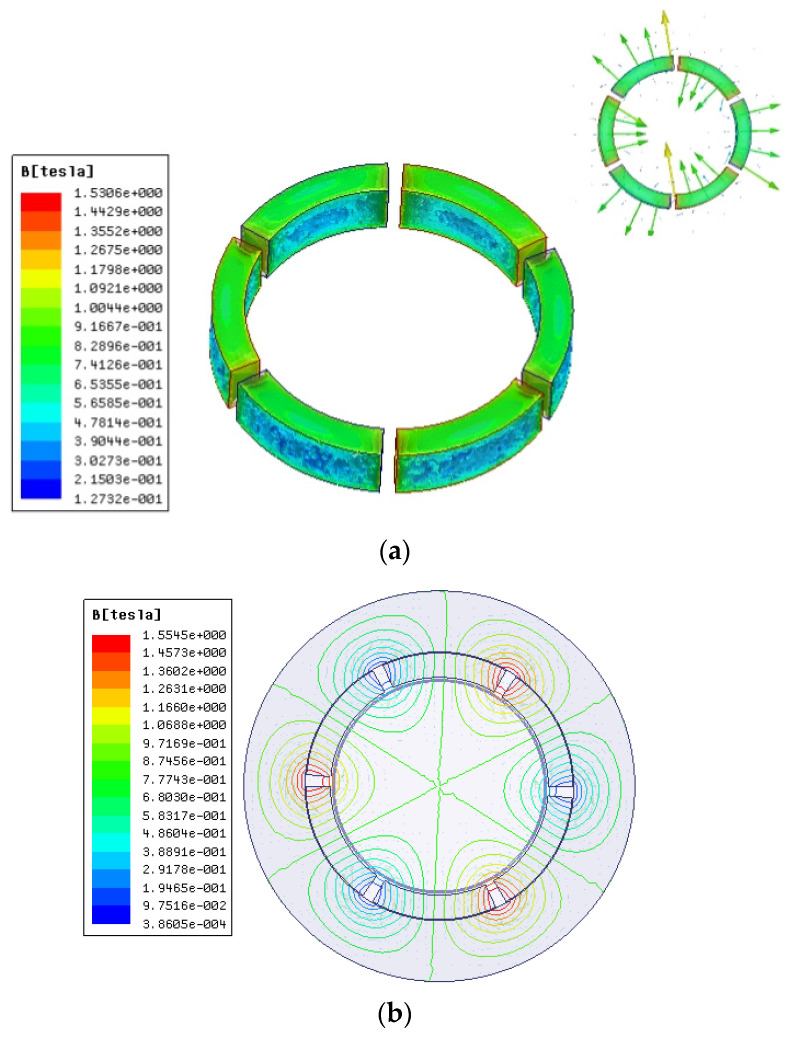

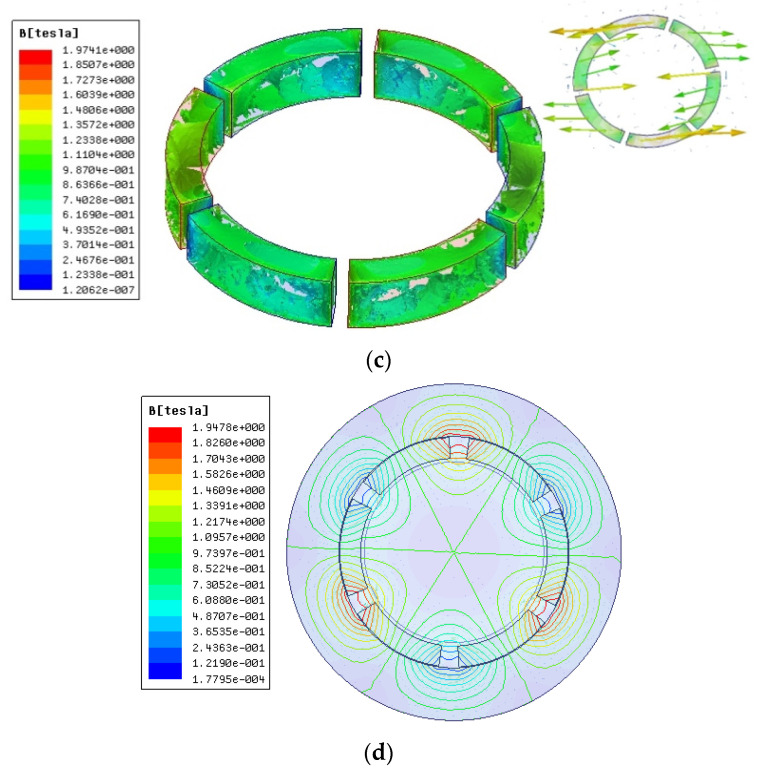
Magnetization modes: (**a**) radial magnetic dense cloud diagram; (**b**) radial magnetic line distribution; (**c**) parallel magnetizing dense cloud diagram; and (**d**) parallel magnetizing force line.

Parallel and radial magnetization are usually used for spherical rotors. Radial and parallel magnetization of permanent magnets are performed by the MAXWELL software. The simulation results of the magnetic flux density and the magnetic line in Figure 5 show that the magnetic field intensity of each magnet increases from the center to the outer edge of the magnet in the radial magnetization mode. The maximum value appears at the junction of the N and S poles of the two permanent magnets, and the magnetic lines of each pole are evenly distributed. For parallel magnetization, the distribution of magnetic field strength and magnetic line is not uniform, so radial magnetization is used for permanent magnets.

**Figure 6:**
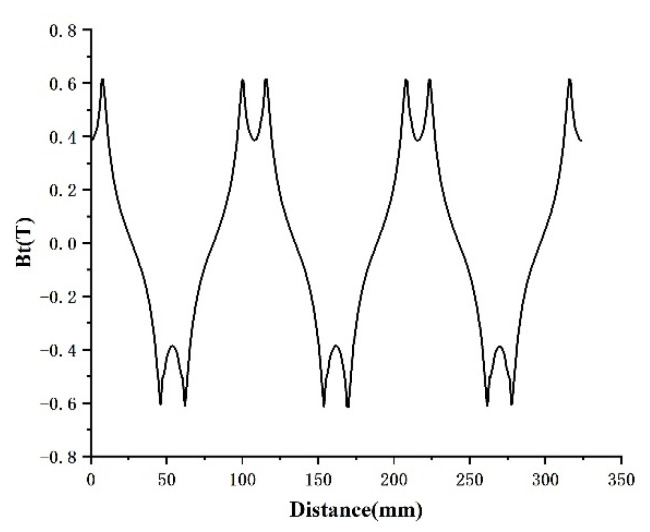
Circumferential air gap density component.

**Figure 7:**
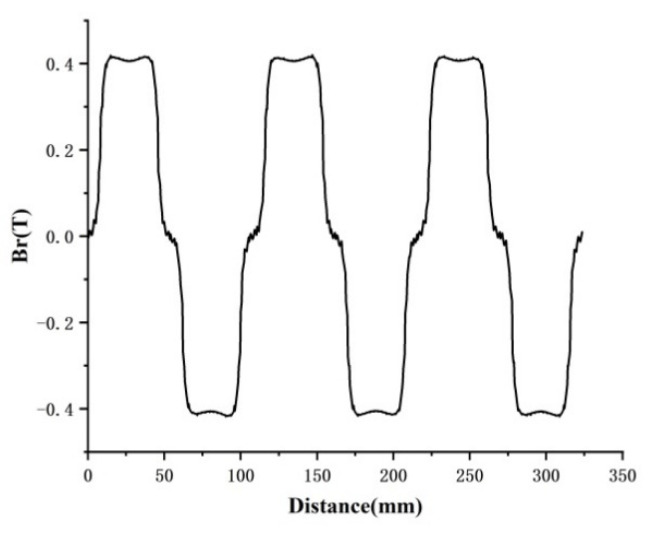
Radial air gap magnetic density component.

In Figure 6 and Figure 7, the component variation in air gap magnetic density under radial magnetization is analyzed, which is composed of three peaks and valleys. The design of the motor is reasonable. Because of the existence of harmonics, the waveform appears as a flat-top wave near the sinusoidal wave. It can be seen from the graph that the gap density reaches its maximum value at the N and S alternation; that is, the utilization of a magnetic field is strong here [10,11]. 

### 3.2. Effect of Permanent Magnet Thickness

**Figure 8:**
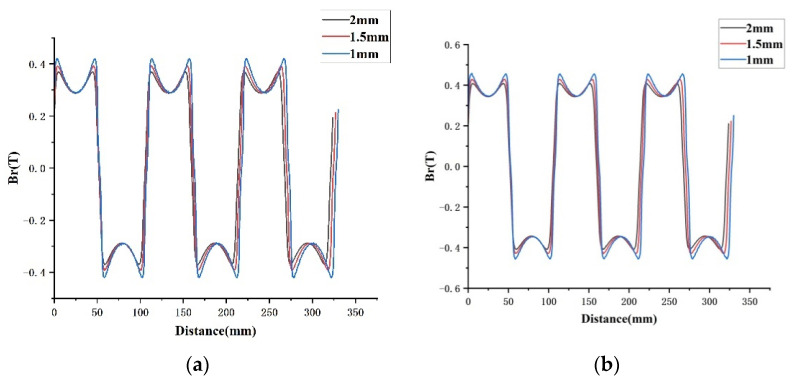

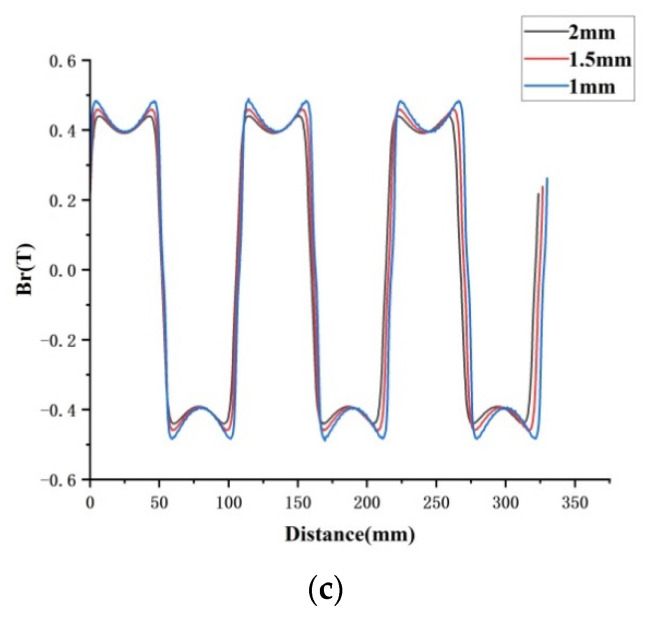
The influence of different thicknesses of permanent magnets on the radial component of air gap magnetic density: (**a**) 8 mm, (**b**) 10 mm, and (**c**) 12 mm.

In the finite element simulation model, the permanent magnet is charged radially, and the gap magnetic field is analyzed by changing the thickness of the permanent magnet to explore the relationship between the two. In Figure 8, the radial component of the air gap magnetic density corresponds (Br) to 8 mm, 10 mm, and 12 mm thickness of the permanent magnets, and magnetic densities at different locations from the permanent magnets are analyzed. The waveform is a flat-top wave, similar to a sine function, with three peaks and three valleys. The simulation results show that the closer the distance to the permanent magnet, the larger the gap magnetic density value, and the thicker the permanent magnet, the larger the gap magnetic density value. When the permanent magnet is 12 mm, the gap magnetic density waveform is closer to the flat-top wave, and the gap magnetic density value reaches 0.4844T at 1 mm from the permanent magnet.

## 4. Alveolar Torque

The Alveolar Torque of Permanent Magnet Motor refers to the torque generated by the interaction of the magnetic field, which is generated by the permanent magnet and the armature alveolar when the armature winding is open [12,13,14,15,16,17]. It will make the motor produce vibration and noise, and speed fluctuation will occur, which will affect the stability of the motor. Therefore, it should be considered as an important factor in the design of the motor. Alveolar torque changes periodically. The smallest common multiple of the motor pole number and the number of molecule slots is the alveolar period. The bigger the period number, the smaller the amplitude of the alveolar torque. It can be expressed as:

(6) $$N_{p}=\frac{LCD\left(Z,\;2p\right)}{Z}$$

where *Np* is the period number, *LCD*(*Z,* 2*p*) is the smallest common multiple of *Z* (stator slot number), and 2*p* is the polar logarithm.

**Figure 9:**
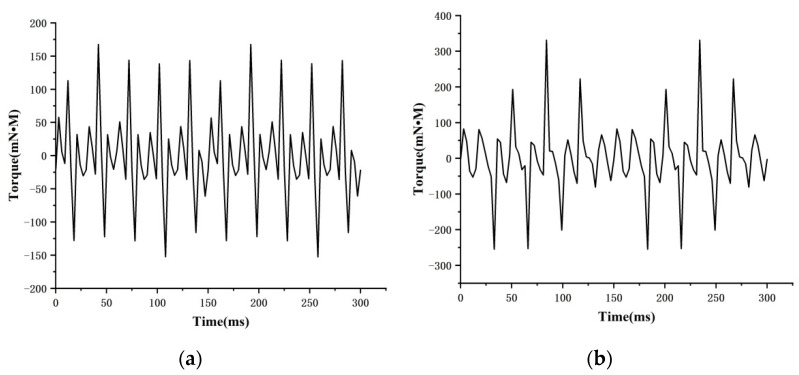
Alveolar torque analysis: (**a**) 6 poles and 15 slots, and (**b**) 6 poles and 18 slots.

The slot torques of the 6-pole 15-slot and the 6-pole 18-slot motors are analyzed. The results in Figure 9 show that the maximum slot torque of the 6-pole 15-slot motor model is 167.6038 mN•M, and that of the 6-pole 18-slot motor is 331.2324 mN•M. The simulation results conform to theoretical knowledge. To ensure good output performance and smooth operation, a 6-pole 15-slot configuration is selected.

## 5. Stator Mode

**Figure 10:**
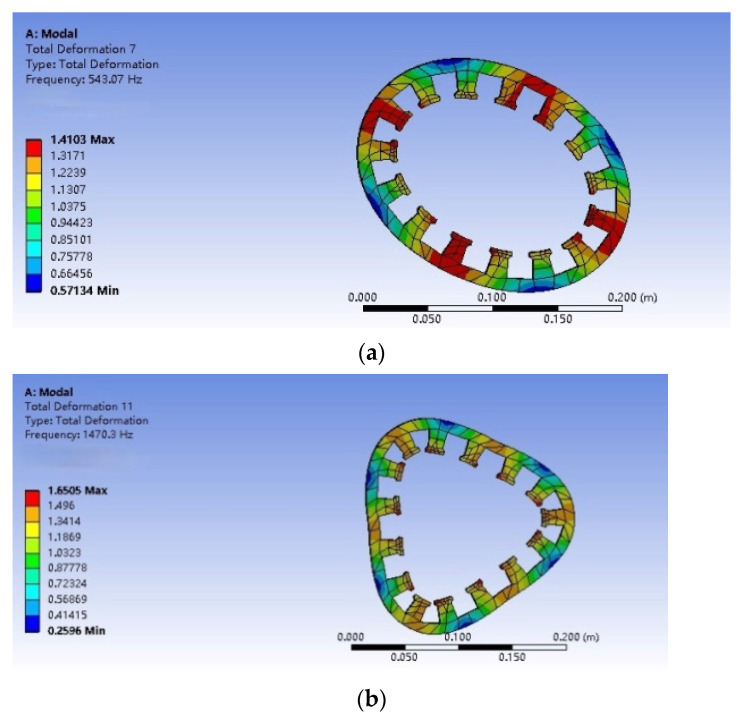

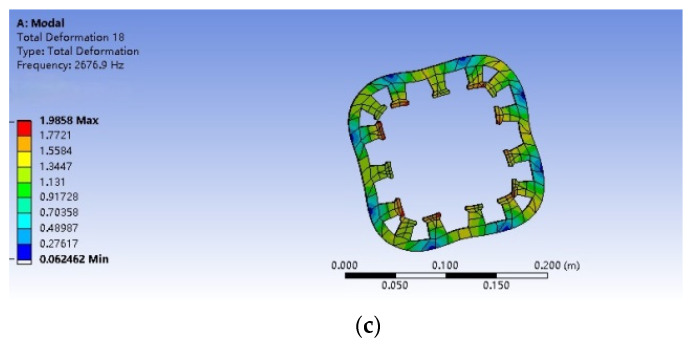
Electromagnetic stator modes: (**a**) second-order mode; (**b**) third-order mode; and (**c**) fourth-order mode.

**Figure 11:**
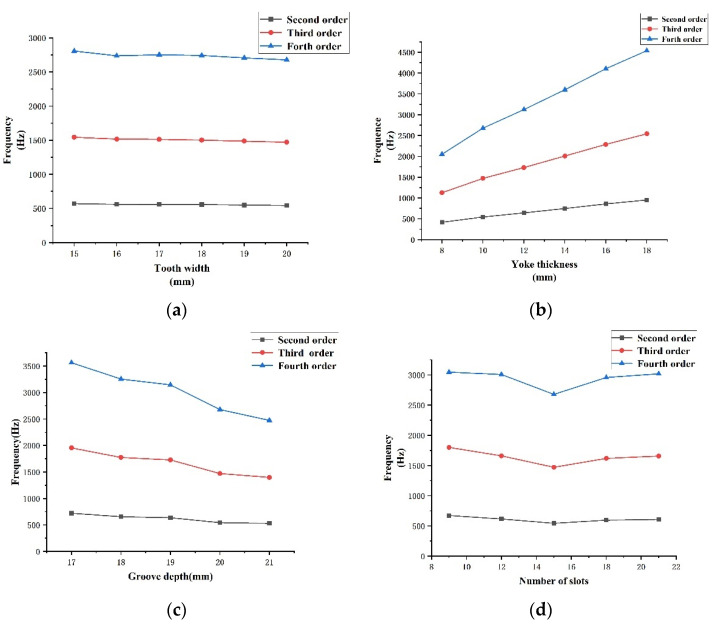
Frequency variation in stator modes with different parameters: (**a**) tooth width; (**b**) yoke thickness; (**c**) number of slots; and (**d**) slot depth.

By analyzing the mode of the motor stator, the structure and size of the stator can be optimized, and the result of the mode frequency can also be used as a criterion to evaluate the performance of the motor. A radial mode analysis of the stator is performed by using the WORKBENCH software. The material parameters are set in Table 2 below. No excitation or constraint is required for the mode simulation. The model is set to the free vibration mode, and the mesh partitioning used the system’s own automatic partitioning mode for the entire selection. The radial modes of the second-, third-, and fourth-order motor stators are shown in Figure 10. By changing the parameters of tooth width, yoke thickness, slot depth, and the number of slots of the stator, the data shown in Figure 11 can be obtained. As shown in Figure 11a, with the increase in tooth width, the mode frequencies of each order show a downward trend, with a higher order and a more obvious downward trend. Figure 11b shows that, as the thickness of the stator yoke increases, the mode frequency grows rapidly and the higher-order frequency increases sharply. In Figure 11c, the groove depth increases and the mode frequency decreases too quickly. Figure 11d shows that, as the number of slots increases, the stator mode frequency decreases first and then increases. The stator mode frequency should not be too large, as the greater the vibration amplitude, the louder the motor noise will be, and wear will be aggravated. This will affect the life of the motor, as well as the motor performance. The final tooth width is 20 mm; yoke thickness is 10 mm; and slot depth is 20 mm. Fifteen slots are used as a reference standard to design motor stators.

The stator model can be simplified into a double-ring model. The force acts on the teeth of the stator first and then on the yoke. The radial mode frequency of the stator is calculated by an electromechanical analogy with the expression [18]:

(7) $$F_{n}=\frac{n\left(n^{2}-1\right)}{\sqrt{n^{2}+1}}\sqrt{\frac{\frac{E_{1}}{R_{1}^{3}}h_{1}^{3}lF_{n1}^{2}+\frac{E_{1}}{R_{2}^{3}}h_{2}^{3}lF_{n2}^{2}}{24\pi \left(m_{1}+m_{2}\right)}}$$

In this formula, *n* represents the order of vibration, *E* is the modulus of elasticity, and the unit is Pa. *H* is the thickness of the ring in mm; *L* is the axis length of the annulus in mm; *m* is mass in kg; *R* is the mean radius of the circle in mm; and *Fn* is a function of the ratio of yoke thickness to mean radius, ranging from 0.96 to 0.98.

## 6. Motor Torque Analysis

The electromagnetic torque of the motor can be calculated by first calculating the value of the torque generated by a single coil, then superimposing and summing up the total electromagnetic torque [19,20,21,22]. According to Lorenz’s Law of Force [23,24], the element of any coil is the torque element produced by it:

(8) $$dT_{c}=r\times F=re_{r}\times \left[B\left(r,\theta ,\phi \right)\times \left(-J_{c}rdrd\delta dl\right)\right]$$

where, *J_c_* is the cross-section current density of powered winding.

**Figure 12:**
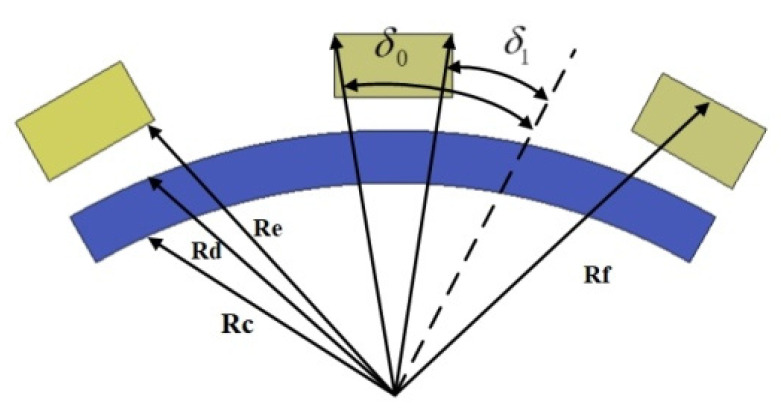
Diagram of equivalent correspondence between the permanent magnet and the coil.

In Figure 12, *R_e_* and *R_f_* are the distances from the center to the inner and outer diameter of the coil, respectively. *δ*_0_ and *δ*_1_ indicate the angle from the coil center line to the coil side, which is calculated by the integral calculation of the torque element.

(9) $$T_{c}=-J_{c}\int_{R_{e}}^{R_{f}}\int_{\delta_{0}}^{\delta_{1}}\left\{\int_{L}re_{r}\times \left[B\left(r,\theta ,\phi \right)\times dl\right]\right\}rdrd\delta$$

Converts the rotation and deflection component torques in spherical coordinates into rectangular coordinates:

(10) $$\left\{ \begin{array}{l} \cos\phi =\frac{x}{r\sin\theta }\\ \sin\phi =\frac{y}{r\sin\theta } \end{array}\right.$$

The expressions for each unit component of the coil are:

(11) $$\left\{ \begin{array}{l} x=r\cos\delta \sin\theta_{c}\cos\phi_{c}-r\sin\delta \cos\varphi \cos\theta_{c}\cos\phi_{c}+r\sin\delta \sin\varphi \sin\phi_{c}\\ y=r\cos\delta \sin\theta_{c}\sin\phi_{c}-r\sin\delta \cos\varphi \cos\theta_{c}\sin\phi_{c}-r\sin\delta \sin\varphi \cos\phi_{c}\\ z=r\cos\delta \cos\theta_{c}+r\sin\delta \cos\phi \sin\theta_{c} \end{array}\right.$$

Introducing unit vectors *e_ΦC_*, *e_θC_*: the two directions are the tangent direction of the equator and the tangent direction of the vertical equator, the former generating a self-rotating moment and the latter generating a deflection moment. The expression of coil element in the Cartesian coordinate system is:

(12) $$dl=r\sin\delta d\phi \left(\cos\phi e_{\varphi c}-\sin\phi e_{\theta c}\right)$$

The rotation torque and the deflection torque of the coil can be obtained simultaneously:

(13) $$T_{ce_{\varphi c}}=J_{c}\int_{R_{e}}^{R_{f}}\int_{\delta_{0}}^{\delta_{1}}\int_{0}^{2\pi }B\left(r,\theta_{c},\phi_{c},\varphi ,\delta \right)\sin\delta \sin\varphi d\varphi drd\delta$$

(14) $$T_{ce_{\theta c}}=J_{c}\int_{R_{e}}^{R_{f}}\int_{\delta_{0}}^{\delta_{1}}\int_{0}^{2\pi }B\left(r,\theta_{c},\phi_{c},\varphi ,\delta \right)\cos\delta \cos\varphi d\varphi drd\delta$$

**Figure 13:**
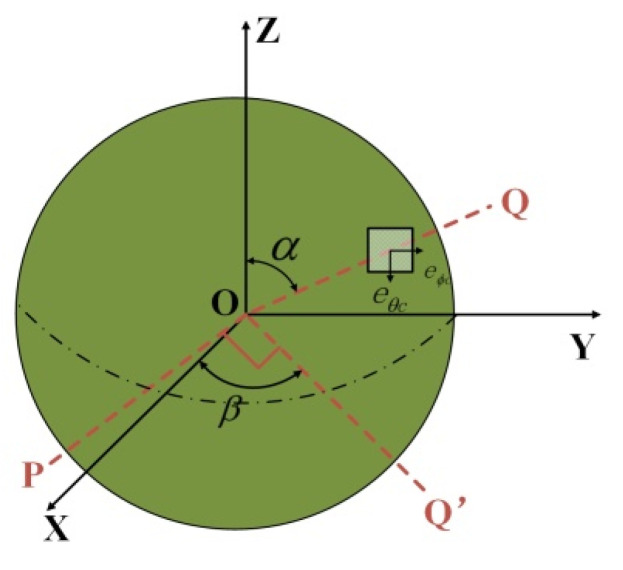
Torque decomposition.

The torque is decomposed as shown in Figure 13, and the angle between OX and OQ is *β*.If the angle between OP and OX is 90° − *β*, then the component of the torque rotating around the *x*-axis, *y*-axis, and *z*-axis is:

(15) $$T_{a}=\left[\begin{array}{l} T_{x}\\ T_{y}\\ T_{z} \end{array}\right]=\left[\begin{array}{c} T_{ce_{\varphi c}}\times \cos\left(90-\beta \right)\\ T_{ce_{\varphi c}}\times \cos\beta \\ T_{ce_{\varphi c}} \end{array}\right]$$

The total electromagnetic torque of the motor can be obtained by summing the torques of a single coil:

(16) $$T_{a1}=\left[\begin{array}{l} T_{x1}\\ T_{y1}\\ T_{z1} \end{array}\right]=\left[\begin{array}{c} \overset{30}{\underset{c=1}{\Sigma }}T_{ce_{\varphi c}}\times \cos\left(90-\beta \right)\\ \overset{30}{\underset{c=1}{\Sigma }}T_{ce_{\varphi c}}\times \cos\beta \\ \overset{30}{\underset{c=1}{\Sigma }}T_{ce_{\varphi c}} \end{array}\right]$$

## 7. Experiment

**Figure 14:**
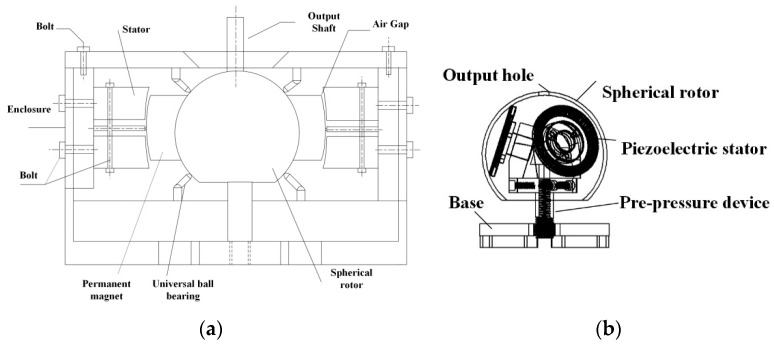
Machining drawing: (**a**) the electromagnetic part and (**b**) the piezoelectric part.

Figure 14 is a mechanical drawing of an electric machine. The external electromagnetic stator is pressed by a silicon steel sheet and has two layers, each with 15 independently controlled coils. The permanent magnet is made of neodymium iron boron with a gap of 0.5 mm between the stator, and the housing is fixed by bolts. The internal piezoelectric structure consists of three identical stators, a spherical rotor, a pre-pressure device, and a base. The stator is made of an elastic base and piezoelectric ceramic patches. The spherical rotor reserves the position of the output shaft, and four friction-free universal ball bearings support the rotator to reduce the weight and keep it running steadily.

**Figure 15:**
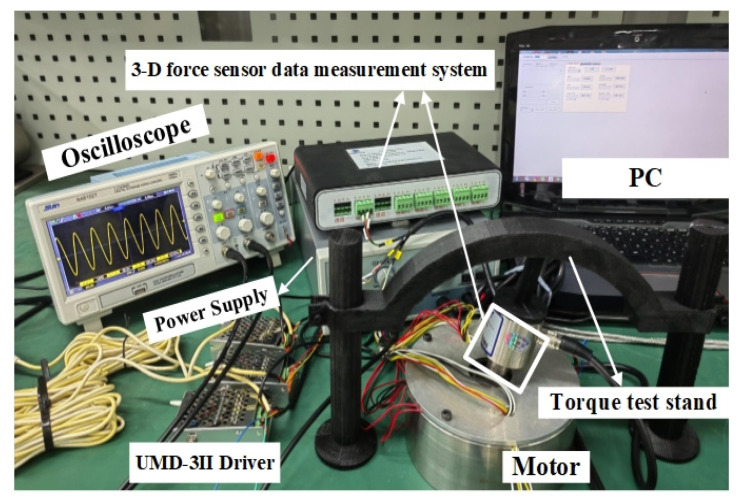
Torque test platform.

In order to verify the rationality of the design of the motor structure and to test the characteristics of the motor, an experimental platform is set up, as shown in Figure 15. In the experiment, UMD-3II, RS232 serial port communication, is used as the driver; a 380 V AC power supply is connected to the power interface of the driver, and a serial port communication line is connected to PC. The sinusoidal and cosine phases A and B are connected to the piezoelectric ceramics.

**Figure 16:**
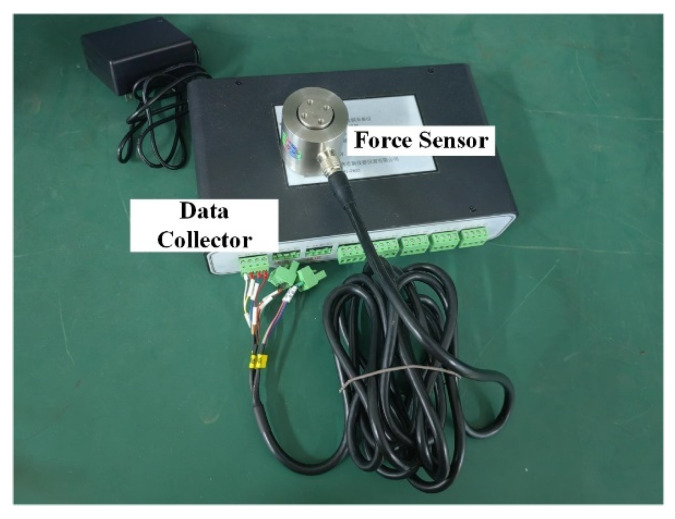
**The** 3D force sensor data measurement system.

**Figure 17:**
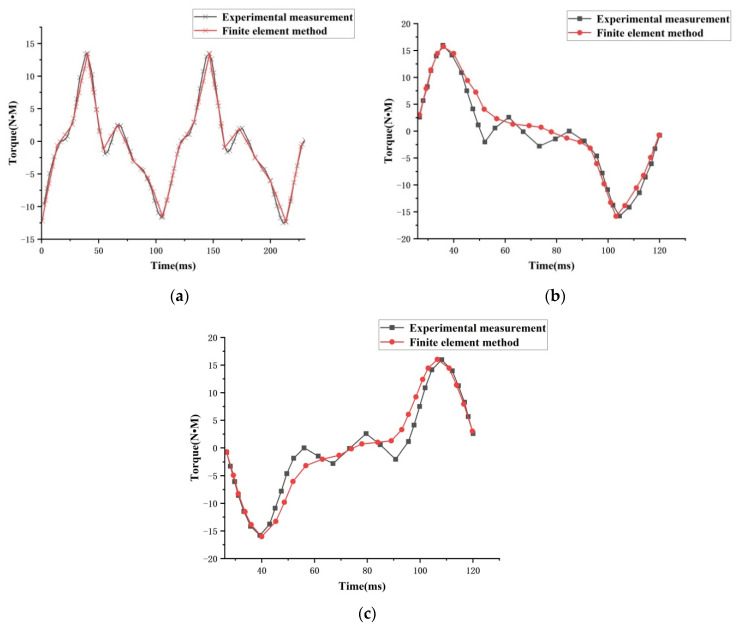
Mixed torque test results: (**a**) forward torque; (**b**) *x*-axis torque; and (**c**) *y*-axis torque.

When testing the motor’s torque performance, the torque measuring bracket designed in this laboratory is fixed with a three-dimensional force sensor by bolts on the output shaft of the hybrid drive motor. The force sensor is connected with an eight-channel data collector to form a three-dimensional force sensor data measurement system, as shown in Figure 16. Through different connection lines, the motor can be transferred to the *x*-axis, *y*-axis, and *z*-axis torque via the data transmission computer. The experimental data of the *z*-axis, *x*-axis, and *y*-axis can be seen in Figure 17, where positive torque and deflection of the hybrid drive motor are in good agreement with the simulation results. Because of the actual manufacturing process and processing accuracy of the motor, there are some errors between the two. The maximum positive torque value in the diagram is 13.38 N•M, and the maximum deflection torque can reach 16.027 N•M.

**Figure 18:**
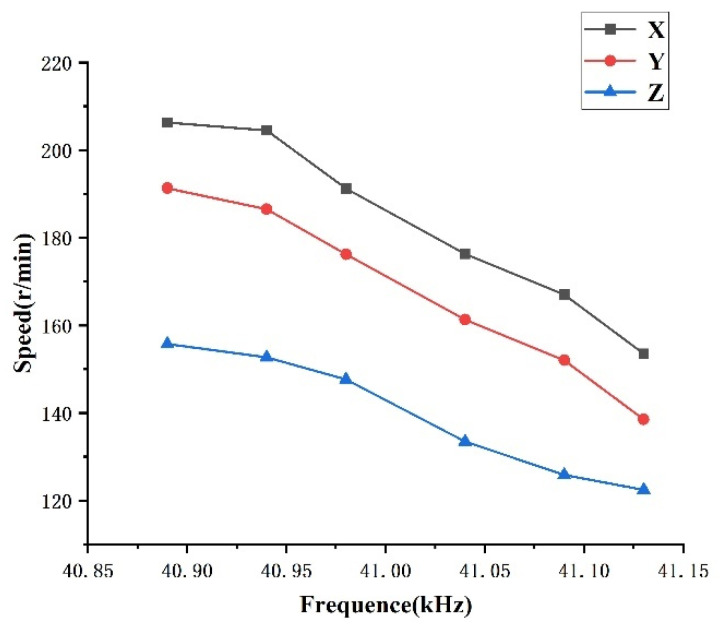
Mixed speed.

To test the speed of the hybrid drive motor, a wireless attitude sensor must be built in the motor rotor. The results are shown in Figure 18. The motor speed decreases with the increase in voltage excitation frequency.

Through the above experiments, the rationality of the structural design of a hybrid drive motor is verified, which provides a new idea for further research on electromagnetic–piezoelectric hybrid drive motors.

## 8. Conclusions

In this paper, the new structure of an electromagnetic–piezoelectric hybrid drive motor is analyzed. Two different ways of magnetization and the effect of the thickness of the permanent magnet on the air gap magnetic density are compared, and the cogging torque of motors with different slot numbers are compared. The optimum air gap magnetic density is determined when the thickness of the permanent magnet is 12 mm and radial magnetization is used, and the motor is designed with 6 poles and 15 slots. Then, the effect of different parameters of the stator on the mode frequency is analyzed by simulation. The motor’s torque is analyzed and simulated, and the prototype’s torque and speed performance are tested by an experiment. The result shows that the torque can reach 16.027 N•M, and the maximum speed is 206.3 r/min, which verifies the advantages of the combination of the two driving modes and the rationality of the motor structure design.

## Figures and Tables

**Table 1:** Electromagnetic structure parameters.

Electromagnetic Stator		Permanent Magnet	
Stator outer diameter	132 mm	Height	22.5 mm
Stator inner diameter	192 mm	Thickness	12 mm
Single-layer stator height	15 mm	Air gap spacing	0.5 mm
Two-layer stator spacing	6 mm	Corresponding angle	51°

**Table 2:** Stator core material parameters.

Density	Elastic Modulus	Shear Modulus	Poisson Ratio
7600 kg/m^3^	E_X_ = E_Y_ = 195 GPa	G_XZ_ = G_YZ_ = 76.9 GPa	0.3
	E_Z_ = 90 GPa	G_XY_ = 34.6 GPa	
